# Physical activity levels of children and adolescents with moderate‐to‐severe intellectual disability

**DOI:** 10.1111/jar.12515

**Published:** 2018-07-11

**Authors:** Marieke Wouters, Heleen M. Evenhuis, Thessa I. M. Hilgenkamp

**Affiliations:** ^1^ Reinaerde Utrecht The Netherlands; ^2^ Department of General Practice Intellectual Disability Medicine Erasmus Medical Center Rotterdam The Netherlands; ^3^ Department of Kinesiology and Nutrition University of Illinois at Chicago Chicago Illinois

**Keywords:** accelerometry, intellectual disability, moderate‐to‐vigorous physical activity, motor development, physical activity

## Abstract

**Background:**

Regular participation of children and adolescents with intellectual disabilites in physical activity is important to maintain good health and to acquire motor skills. The aim of this study was to investigate the habitual physical activity in these children.

**Methods:**

Sixty‐eight children and adolescents (2–18 years) with a moderate‐to‐severe intellectual disability were included in the analyses. They wore an accelerometer on eight consecutive days. Data was analysed by use of descriptive statistics and multiple linear regression analyses.

**Results:**

The participants took on average 6,677 ± 2,600 steps per day, with intensity of 1,040 ± 431 counts per minute. In total, 47% of the participants were meeting physical activity recommendations. Low motor development was associated with low physical activity.

**Conclusions:**

As more than half of the participants were not meeting the recommendations, family and caregivers of these children should focus on supporting and motivating them to explore and expand their physical activities.

## 1 INTRODUCTION

Physical and mental health benefits of physical activity in childhood and adolescence are well known (Boreham & McKay, 2011; Boreham &Riddoch, 2001; Hartman, Houwen, Scherder, & Visscher, 2010; Janssen & Leblanc, 2010; Loprinzi, Cardinal, Loprinzi, & Lee, 2012; Warburton, Nicol, & Bredin, 2006). Physical activity is also critical to acquire motor skills (Loprinzi et al., 2012) such as running and jumping, that are important to remain physically active and fit over time (Loprinzi et al., 2012; Stodden et al., 2008). The World Health Organization (WHO) recommendation for healthy physical activity behaviour for children and adolescents is at least 60 min of moderate‐to‐vigorous physical activity (MVPA) every day (WHO, 2010), which is comparable to approximately 12,000 steps per day (Colley, Janssen, & Tremblay, 2012). The positive effects of physical activity are even more important for children and adolescents with intellectual disabilities, as they have more health and motor problems (Oeseburg, Dijkstra, Groothoff, Reijneveld, & Jansen, 2011), less physical fitness (Hartman, Smith, Westendorp, & Visscher, 2015; Salaun & Berthouze‐Aranda, 2012; Wouters, Evenhuis, & Hilgenkamp, Submitted) and less developed motor skills than typically developing (TD) peers (Hartman et al., 2010; Pereira, Basso, Lindquist, da Silva, & Tudella, 2013; Rintala & Loovis, 2013; Vuijk, Hartman, Scherder, & Visscher, 2010).

Previous research on the physical activity behaviour of individuals with intellectual disability, measured with accelerometers, showed that children and adolescents with intellectual disability were less active than TD children and adolescents (Einarsson, Johannsson, Daly, & Arngrimsson, 2016; Einarsson et al., 2015; Foley, Bryan, & McCubbin, 2008; Frey, Stanish, & Temple, 2008; Hinckson & Curtis, 2013). Published percentages of children and adolescents with intellectual disability complying with the physical activity recommendations vary considerably from 0 to 42% (Downs, Fairclough, Knowles, & Boddy, 2016; Einarsson et al., 2015, 2016; Leung, Siebert, & Yun, 2017; Shields, Dodd, & Abblitt, 2009).

Within previous research, physical activity levels differed among subgroups. Several studies, both in TD children and in children with intellectual disability, found a negative association between age and volume and/or intensity of physical activity (Cooper et al., 2015; Dumith, Gigante, Domingues, & Kohl, 2011; Esposito, MacDonald, Hornyak, & Ulrich, 2012; Izquierdo‐Gomez et al., 2014). Other studies did not find any age effect (Downs et al., 2016; Foley et al., 2008; Izquierdo‐Gomez et al., 2017; Van Der Horst, Paw, Twisk, & Van Mechelen, 2007).

As in TD children and adolescents, sex is an important covariate for the volume of physical activity. Boys with intellectual disability were more active than girls (Foley et al., 2008; Izquierdo‐Gomez et al., 2014, 2017; Phillips & Holland, 2011). Furthermore, children and adolescents with Down syndrome (DS) were less active than their peers with other causes of intellectual disability (Phillips & Holland, 2011), which makes DS an important covariate too. The difference between boys and girls, and children and adolescents with and without DS, might be due to the difference in motor development. Girls with intellectual disability have less developed motor skills than boys with intellectual disability (Rintala & Loovis, 2013; Simons et al., 2008; Westendorp et al., 2014), and children and adolescents with DS have a greater delay in motor development than their peers with other causes of intellectual disability (Connolly & Michael, 1986; Parikh, Kulkarni, Abraham, Rao, & Khatri, 2013). As far as the present authors know, the association between motor development and the volume of physical activity has never been studied in children and adolescents with intellectual disability, while this would give potential directions for interventions to improve the physical activity in this specific population.

Studies in adults and older people show that physical activity levels decrease with increasing severity of intellectual disability (Hilgenkamp, van Wijck, & Evenhuis, 2012; Phillips & Holland, 2011), but no information is available on this association in children and adolescents with intellectual disability. Furthermore, little is known on the habitual physical activity levels of children and adolescents with more severe levels of intellectual disability, as the majority of previous studies were conducted in children and adolescents with mild‐to‐moderate intellectual disability (Leung et al., 2017). Only few studies included children or adolescents with severe intellectual disability. One study focused only on physical activity during physical education and recess (Pan, Liu, Chung, & Hsu, 2014). In this study, adolescents with intellectual disability spent less time in MVPA during recess than their TD peers. In a more recent study (Downs et al., 2016), 24% of participants (5–15 years) from special education schools with moderate‐to‐severe learning disabilities were reaching the physical activity recommendation and their mean habitual MVPA was 49 min per day. Another study showed even lower rates of daily MVPA: only 5% of Icelandic children and adolescents with mild‐to‐severe intellectual disability (6–16 years) were achieving the recommendations of 60‐min MVPA every day (Einarsson et al., 2016).

Even though these studies give us an idea of the volume of habitual physical activity of children and adolescents with more severe intellectual disability, important information is missing. No subanalyses were performed on the level of intellectual disability, and motor development was not assessed. Moreover, in these previous studies, cut‐points based on energy expenditure data of TD children and adolescents were used to classify MVPA. However, it is likely that the energy expenditure of individuals with intellectual disability is higher than that of the general population during tasks like walking (Agiovlasitis, McCubbin, Yun, Pavol, & Widrick, 2009; Lante, Reece, & Walkley, 2010), possibly caused by autonomic dysfunction (Fernhall, Mendonca, & Baynard, 2013) and different gait patterns (Almuhtaseb, Oppewal, & Hilgenkamp, 2014). Therefore, cut‐points based on the general population might not be representative for individuals with intellectual disability. This has been confirmed by McGarty, Penpraze, and Melville (2016), who developed and validated specific cut‐points for children and adolescents with intellectual disability (8–11 years) against direct observation. McGarty's cut‐points differ fairly from the cut‐points for TD children like those from Evenson, Catellier, Gill, Ondrak, and McMurray (2008) as can be seen in Table 1. It is therefore likely that use of cut‐points for TD children will lead to underestimation of MVPA in children and adolescents with intellectual disability.

**Table 1:** Cut‐points to classify the intensity of physical activity based on counts per minute (cpm)

	Evenson et al.	McGarty et al.	Vector magnitude
	Vertical axis	Vertical axis	
Sedentary	≤100	≤507	≤1,863
Light intensity	100–2,295	508–1,007	1,864–2,609
Moderate intensity	2,296–4,011	1,008–2,300	2,610–4,214
Vigorous intensity	≥4,012	≥2,301	≥4,215

Based on the above, the present authors conclude that information is needed on habitual physical activity of children and adolescents with more severe intellectual disability. The lack of this knowledge is the more problematic as these individuals are likely to be at a higher risk of chronic health conditions than their peers with less severe intellectual disability (Moss, Goldberg, Patel, & Wilkin, 1993; van Schrojenstein Lantman‐de Valk et al., 1997). More information on the volume and intensity of physical activity and child characteristics associated with low physical activity will help professionals and researchers developing and targeting interventions to increase the physical activity of children and adolescents with moderate‐to‐severe intellectual disability.

Therefore, the following questions were to be answered in the current study: (a) What is the volume and intensity of PA of children and adolescents with moderate‐to‐severe intellectual disability?; (b) How many participants are active enough to reach the physical activity recommendations of 60‐min MVPA per day?; and (c) Which child characteristics (age, sex, level of intellectual disability, DS, motor development) are associated with physical activity outcomes?

## 2 METHODS

### 2.1 Participants

Children aged 2–18 years with a moderate‐to‐severe intellectual disability who were able to walk independently were invited to participate in this cross‐sectional study, which was part of a larger study focusing on physical fitness. All potential participants received care or support in one of seven specialized day program facilities of a service provider for people with disabilities in the Netherlands. These day program facilities are specialized to support children and adolescents with intellectual disability that are unable to go to a mainstream or special school, due to their severe developmental delay or additional medical or behavioural comorbidity.

Suitability to participate in the study with regard to the level of intellectual disability was performed by the behavioural therapist or psychologist of the child, based on available psychological test results (moderate intellectual disability: IQ 40–55; severe intellectual disability: IQ 20–40). Parents or legal representatives of the children and adolescents who met the inclusion criteria received an invitation letter. Children were included in the study after their parents or legal representatives had signed the informed consent form.

Ethical approval was obtained (MEC‐2013‐491) from the Ethics Committee of the Erasmus Medical Center. The study adheres to the Declaration of Helsinki for research involving human subjects (World Medical Association, 2013).

### 2.2 Physical activity assessment

Physical activity was measured with triaxial accelerometers, Actigraph GT3x+ (Manufacturing Technologies Inc.). These devices translate movement in the direction of three internal axes into counts. Actigraphs have been validated in TD children and adolescents (De Vries, Bakker, Hopman‐Rock, Hirasing, & van Mechelen, 2006; De Vries et al., 2009), children and adolescents with physical disabilities (Clanchy, Tweedy, Boyd, & Trost, 2011) and with intellectual disability (McGarty et al., 2016). Participants were asked to wear the accelerometer on the right hip, by use of an elastic belt. Their parents or caregivers were instructed to let the child wear it continuously on eight consecutive days during waking hours, except during water‐based activities like showering and swimming. Parents or caregivers were asked to record special events like sickness on a standardized sheet. On this sheet, activities such as swimming and cycling could also be recorded. The present authors did not include these outcomes in the analysis, because of a large number of missing values.

### 2.3 Data processing

Data were sampled with a frequency of 30 Hz. Raw data were acquired in 15‐s time sampling intervals (epochs). A 15‐s epoch was selected, because of the fragmentary nature of children's physical activity (Cliff, Reilly, & Okely, 2009; Reilly et al., 2008).

Non‐wear time was defined as ≥20 min of consecutive zeros, with no allowance of epochs with counts above zero (Esliger, Copeland, Barnes, & Tremblay, 2005). In studies with TD children, 10‐ and 20‐min strings of zeros are the most common (Cain, Sallis, Conway, Van Dyck, & Calhoon, 2013; Cliff et al., 2009; Esliger et al., 2005; Janssen et al., 2015). In previous studies with children and adolescents with intellectual disability, strings of consecutive zeros of 10 min (Phillips & Holland, 2011), 30 min (Einarsson et al., 2016) and 60 min (Izquierdo‐Gomez et al., 2014) were used.

Non‐wear time was excluded from analysis. Data with at least 4 days of recording, with a minimum of 480 registered minutes (8 hr) per day were included in the analysis, as this is said to have a reliability of 91%–92% (Rich et al., 2013). No distinction was made between week or weekend days, as no significant differences were found between the physical activity on week or weekend days (data not shown).

Total volume of daily PA was expressed as steps per day. The overall activity level was calculated by summation of counts and expressed as counts per minute (cpm). Higher cpm means greater activity intensity. The intensity of physical activity was categorized as sedentary behaviour, light, moderate and vigorous activity based on specific vector magnitude (VM) cut‐points established in children with intellectual disability (McGarty et al., 2016) (Table 1). To compare the outcomes with previous and future studies, cpm based on the vertical axis, and intensity derived with Evensons’ cut‐points (Evenson et al., 2008) are also presented in Table 3.

Total time spent in the different categories of intensity was expressed in minutes and as a percentage of total daily wear time. Total time spent in moderate‐to‐vigorous physical activity (MVPA) was calculated by summing the time spent in moderate and vigorous intensity.

### 2.4 Motor development

The gross motor scale of the Bayley Scale of Infant and Toddler Development, third edition (BSID‐III) (Bayley, 2006) was completed by physical therapists and was used to give insight into the gross motor development. The BSID is designed to measure the developmental status of young children and adolescents up to 42 months, but it can also be used to assess the development of individuals with severe delays, such as children and adolescents with intellectual disability (Pearson Education, 2008). A score of 42–43 corresponds to the development of a TD child aged 12 months, a score of 57 to 24 months, 64 points to a 36 months and the maximum score (67–72 points) to 42 months (Bayley, 2006).

### 2.5 Other measurements

Height was measured with a portable stadiometer (Seca 213, Hamburg, Germany), accurate at 0.1 cm level. Body weight was measured using an electronic calibrated scale (Tanita TBF‐300A, Illinois, USA), accurate at 0.1 kg level. The participants were on bare feet and wore light clothes. BMI was calculated as body weight in kg divided by height in meters squared. BMI‐for‐age‐Z scores (zBMI) were calculated according to the WHO Growth references (de Onis et al., 2007; WHO Multicentre Growth Reference Study Group, 2006). Participants were classified as underweight when zBMI was <2 *SD*. Participants at age 0–5 years with BMI >2 *SD* were classified as overweight, >3 *SD* as obese. For older children and adolescents (6–18 years), >1 *SD* was classified as overweight, and >2 *SD* as obese (de Onis & Lobstein, 2010).

Information on autism spectrum disorder (ASD) was provided by the behavioural therapist or psychologist of the participants. Information on chronological age, DS and physical disabilities was extracted from the records of the care provider.

Adaptive behaviour was used as an indicator of the level of intellectual disability, as intellectual disability is characterized by significant limitations both in intellectual functioning and in adaptive behaviour (Schalock et al., 2010). Adaptive behaviour was assessed by the Dutch version of the Vineland Adaptive Behavioural Scale (de Bildt & Kraijer, 2003; Sparrow, Balla, & Cicchetti, 1984). In this scale, three types of skills are covered: conceptual, social and practical skills. The scale was filled in by the caregiver of the child, and scored and converted to relative age score by the own behavioural therapist or physiologist.

### 2.6 Data analysis

Normality of all variables was checked by using Kolmogorov‐Smirnov test, and skewness and kurtosis values.

Children with at least 4 days of eight hour data were selected for data analysis. The characteristics of the participants were compared to the non‐participants (with not enough valid data) to investigate selective drop‐out. For this comparison, *χ* ^2^ statistics and independent *t*‐tests were used, and Mann–Whitney *U* test as non‐parametric alternative.

Descriptive statistics were used to study the wear time and physical activity parameters (steps per day, cpm, minutes MVPA, distribution of physical activity intensities, and the percentage participants reaching physical activity recommendations of daily ≥60 min MVPA). These physical activity parameters were presented for the total sample in Table 3. In the appendix , in Table A1, the results were sorted by, respectively, boys and girls, and children and adolescents with DS and with other causes of intellectual disability.

To find associations of the child characteristics and the physical activity outcomes, linear regression analyses were performed with steps per day, cpm, and minutes MVPA, determined by McGarty's cut‐points as dependent variables. The independent variables were entered in two blocks into the regression analysis (block 1: sex, age, adaptive behaviour, DS; block 2: motor development). For this analysis, the present authors checked the assumptions of multicollinearity with the variance inflation factor (VIF), which needed to be below 10, and with the correlations between the independent variables, which should not contain correlations above 0.8. Homoscedasticity was checked with a plot of regression standardized residual (*ZRESID) against regression standardized predicted value (*ZPRED) (Field, 2009).

Data were processed and analysed using Actilife 6, Excel (Microsoft 2016) and IBM SPSS Statistics 21. Alpha was set at 5%.

## 3 RESULTS

### 3.1 Participants

**Figure 1:**
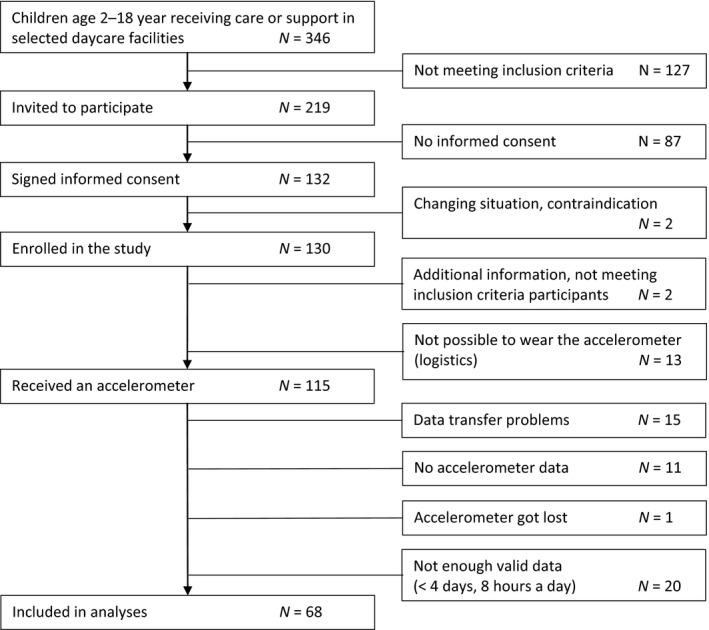
Flow diagram of inclusion process

Of 130 children and adolescents that were included in the study, 68 participants had enough valid accelerometer data to be included in the analysis (Figure 1). The sample consisted of 43 boys and 25 girls, their age ranged between 2 and 18 years. Characteristics of the participants with physical activity data were not significantly different from those of the non‐participants (Table 2). According to the records of the children, two participants had motor disabilities (one cerebral palsy, one scoliosis).

**Table 2:** Characteristics of participants included in the study, and the participants excluded from the study

	Included participants			Excluded participants		
	*n*	%	*M *± *SD*	*n*	%	*M *± *SD*
Total	68	100		60	100	
Sex						
Boys	43	63		40	67	
Girls	25	37		20	33	
Age (years)	68		9.4 ± 4.3	60		9.8 ± 3.8
2–7	28	41		16	27	
8–12	20	29		24	40	
13–18	20	29		20	33	
Level of intellectual disability						
Moderate	30	44		24	40	
Severe	38	57		35	58	
Adaptive behaviour (y)^3^	60		1.9 ± 1.6	51		1.9 ± 0.9
Motor development (score BSID‐III)	68		61 ± 7	50		58 ± 8
Down syndrome						
With	16	24		14	23	
Without	52	76		46	77	
ASD						
With	20	29		24	40	
Without	45	66		35	58	
BMI (kg/m^2^)	62		19.0 ± 4.2	56		19.7 ± 4.6
zBMI	62		0.8 ± 1.3	56		0.9 ± 1.1
Overweight	22	32		25	42	

Notes

No significant difference between participants with and without PA‐data.

*M*, mean; *SD*, standard deviation; PA, physical activity; ASD, autism spectrum disorder; BMI, body mass index.

a As continuous indicator of level of intellectual disability.

### 3.2 Physical activity

The participants wore the accelerometer on 4 to 8 days (mean 6.5 ± 1.3 days, 95% Confidence Interval (CI) = 6.1–6.8). For 58 of the 68 children, at least one weekend day was included. Average wearing time per day was 675 ± 76 min (95%CI = 656–693). For the total group, the volume of physical activity was on average 6,677 ± 2,600 steps per day (95%CI = 6,048–7,306), with an activity level of 1,040 ± 431 VM‐cpm (95%CI = 936–1,144) and 92 ± 46 min of MVPA per day (95%CI = 81–103) using the McGarty's cut‐points (Table 3).

**Table 3:** Descriptive statistics of physical activity parameters (*M* ± *SD*, [95% CI]), analysed with VM‐cut‐points of McGarty et al. and vertical axis‐cut‐points of Evenson et al

	McGarty et al.		Evenson et al.	
	*M* ± *SD*	95% CI	*M* ± *SD*	95% CI
Steps per day	6,677 ± 2,600	6,048–7,306		
Counts per minute	1,040 ± 431	936–1,144	447 ± 244	388–506
MVPA (min)	92 ± 46	81–103	28 ± 20	24–33
Daily ≥60 min MVPA (*n* (%))	32 (47)	35–59	3 (4)	0–9
Distribution of daily physical activity levels (%)				
Sedentary	78 ± 9	76–80	59 ± 11	56–61
Light PA	8 ± 3	7–8	37 ± 9	35–39
Moderate PA	9 ± 4	8–10	3 ± 2	2–3
Vigorous PA	5 ± 4	4–6	1 ± 1	1–2

Note

MVPA, moderate‐to‐vigorous physical activity.

More than three quarters of the day (78%, 530 ± 91 min) were spent sedentary, when using McGarty's cut‐points. The remaining time was spent with light intensity (8%, 53 ± 17 min), moderate intensity (9%, 59 ± 26 min) and vigorous intensity (5%, 33 ± 25 min). Forty‐seven per cent of the participants were active enough to meet the recommendations of at least 60 min of MVPA every day, according to McGarty's cut‐points (Table 3).

The results of the linear regression analysis (Table 4) indicated that the number of steps per day was associated with boys (*β* = −0.33; *p* = 0.01) and having DS (*β* = −0.25; *p* < 0.05) in the first model. However, in model 2 when motor development was added, sex and DS were no longer significant predictors, but motor development was, for all three physical activity parameters (*β* = 0.49–0.52; *p* < 0.01). For cpm, adaptive behaviour became also a significant predictor in the second model (*β* = −0.34, *p* = 0.04).

**Table 4:** Linear regression analysis (*n* = 60) on physical activity outcomes analysed with use of McGarty's cut‐points

	Steps per day					Counts per minute					Minutes MVPA				
	*B*	*SE B*	*β*	*p*	Adj *R* ^2^	*B*	*SE B*	*β*	*p*	Adj *R* ^2^	*B*	*SE B*	*β*	*p*	Adj *R* ^2^
Model 1															
Intercept	9,633	1,168			13%	1,304	212			0%	134.4	21.5			4%
Sex (0 = boy; 1 = girl)	−**1,770**	**682**	**−0.33**	**0.01**		61	124	−0.07	0.62		−20.2	12.5	−0.21	0.11	
Age (year)	−95	80	−0.15	0.24		−15	15	−0.14	0.32		−2.3	1.5	−0.21	0.13	
Adaptive behaviour (year)^6^	359	213	0.22	0.10		−15	39	−0.06	0.69		3.4	3.9	0.12	0.38	
DS (0 = no DS; 1 = DS)	−**1,567**	**779**	**−0.25**	**0.049**		−12	141	−0.01	0.93		4.6	14.3	0.04	0.75	
Model 2															
Intercept	−1,111	3,414			26%	−588	623			12%	−34.4	64.4			15%
Sex (0 = boy; 1 = girl)	−715	703	−0.13	0.31		125	128	0.14	0.33		−3.7	13.3	−0.04	0.78	
Age (year)	−54	75	−0.09	0.48		−7	14	−0.07	0.59		−1.6	1.4	−0.15	0.25	
Adaptive behaviour (year)^6^	−77	236	−0.05	0.74		−**92**	**43**	**−0.34**	**0.04**		−3.4	4.5	−0.12	0.44	
DS (0 = no DS; 1 = DS)	−1,098	730	−0.18	0.14		70	133	0.07	0.60		12.0	13.8	0.11	0.39	
Motor development (score)	**167**	**50**	**0.49**	**<0.01**		**29**	**9**	**0.52**	**<0.01**		**2.7**	**0.9**	**0.44**	**<0.01**	

Notes

DS, down syndrome. The bold values represent the significant values in the regression models.

a As continuous indicator of level of intellectual disability.

## 4 DISCUSSION

This study into the physical activity of 68 children and adolescents with moderate‐to‐severe intellectual disability shows that only 47% is meeting the WHO‐recommendations of at least 60 min of daily moderate‐to‐vigorous physical activity (MVPA) according to intellectual disability‐specific intensity cut‐points. The participants take on average 6,677 ± 2,600 steps per day. The average intensity is 1,040 ± 431 counts per minute (cpm), and the children and adolescents spent 92 ± 46 min per day at moderate‐to‐vigorous intensity. Motor development and adaptive behaviour (as indicator of level of intellectual disability) are the only child characteristic associated with the volume and/or intensity of the physical activity.

The percentage of participants complying with the recommendations of MVPA in the current study (47%) is higher than seen in previous studies in children and adolescents with intellectual disability (0%–42%) (Downs et al., 2016; Einarsson et al., 2016; Leung et al., 2017). This difference may be explained by the different cut‐points used. The present authors were the first to use intellectual disability‐specific cut‐points that differ from cut‐points based on data from typically developing (TD) children and adolescents (Table 1). The present authors did analyse our data with the cut‐points for TD children and adolescents (Evenson et al., 2008), and then, only 4% of the participants was reaching the recommended 60 min of MVPA every day. This illustrates the huge effect cut‐points have on the outcome, and thereby the conclusions drawn.

Even though only one study has been conducted on the intellectual disability‐specific cut‐points (McGarty et al., 2016), it is plausible that individuals with intellectual disability need specific cut‐points, as their energy expenditure during activities like walking is higher (Agiovlasitis et al., 2009; Lante et al., 2010). Further research is definitely needed to cross‐validate these cut‐points, and study children and adolescents in other age groups (<8 years and >11 years) and individuals with specific syndromes like Down syndrome. Furthermore, an intellectual disability‐specific recommendation for steps per day is needed, as the current recommendation is based on the energy expenditure of TD children and adolescents (Colley et al., 2012). This intellectual disability‐specific recommendation will be accessible and relevant for clinical practice, as steps per day captures the imagination of children, parents and care givers, and activity tracking applications on smart phones and smart watches display their outcomes in steps per day.

Comparison of results of different studies is difficult when different measurement methods are being used: type of accelerometers (Actigraph, RT3 and Actical), wear location (waist and lower back) and data processing (epoch, classification of valid data and non‐wear time) differed in previous research. Due to new insights like new types of accelerometers and intellectual disability‐specific cut‐points (McGarty et al., 2016), such differences will continue to exist. Therefore, accurate description of the methods, and presentation of raw data are important to interpret results and make comparison more feasible to clarify differences.

From previous research in children and adolescents with intellectual disability, raw data have hardly been reported. Only Phillips and Holland (2011) reported steps per day: in their small paediatric subgroup (12–15 years; *n* = 7), boys took 7,181 ± 179 steps per day, and girls 6,918 ± 749, which is comparable to the current results (6,677 ± 2,600, Table 3). Counts per minute were more often reported: many studies in youth with intellectual disability showed average cpm between 300 and 450 cpm (Einarsson et al., 2015, 2016; Izquierdo‐Gomez et al., 2014, 2017; Shields, Hussey, Murphy, Gormley, & Hoey, 2015; Shields et al., 2013; Ulrich, Burghardt, Lloyd, Tiernan, & Hornyak, 2011). Phillips and Holland (2011) found higher cpm (680–836 cpm). These cpm were based on vertical activity counts and are not comparable to the cpm‐values based on VM. Therefore, in the current study, the present authors also report the cpm based on vertical activity (447 ± 244 cpm), to be comparable to the outcomes of previous research.

Recent reviews on physical activity interventions performed in children and adolescents with intellectual disability showed that an increase in physical activity is difficult (Frey, Temple, & Stanish, 2017; McGarty, Downs, Melville, & Harris, 2017). In the review by Frey et al. (2017), nine of eleven studies reported a direct positive effect of the intervention on physical activity, and also long‐term positive effects were found in three studies. However, the review also illustrated that there is a lack of studies with good quality focusing on physical activity interventions for youth with intellectual disability. The authors of the review conclude that effective intervention components cannot be concluded from the outcomes. McGarty et al. (2017) concluded in their review of five studies and meta‐analysis of two studies that current interventions are ineffective in increasing physical activity levels. Therefore, research on effective intervention components for this specific population is needed.

One of these components might be motor skills. The current study indicated that participants with low motor development were less physically active than the participants with more developed motor skills. Unfortunately, little is known on the effectiveness of interventions increasing motor skills in children and adolescents with intellectual disability. Based on previous studies, Lucas et al. (2016) and Hocking, McNeil, and Campbell (2016) suggested in their reviews that task‐specific training may be useful, but that the overall quality of evidence is low. More research is needed to study if and how motor development can be increased in children and adolescents with intellectual disability and whether increased motor development positively influences the volume of physical activity directly (in childhood or adolescence) and in future (in adulthood), as is seen in TD children and adolescents (Loprinzi et al., 2012; Stodden et al., 2008).

In the current study, some differences between boys and girls, and children and adolescents with DS and with other causes of intellectual disability were found, but when adding motor development to the regression model, the sex and DS difference were no longer significant (Table 4). This suggests that motor development is at least partly explaining the effect of sex and DS, which is in accordance with previous research that found less motor skills in girls with intellectual disability compared to boys with intellectual disability (Rintala & Loovis, 2013; Simons et al., 2008; Westendorp et al., 2014), and in children and adolescents with DS compared to children and adolescents with other causes of intellectual disability (Connolly & Michael, 1986; Parikh et al., 2013).

The level of intellectual disability was another potential factor influencing the volume of physical activity, but until now, it was mainly studied in adults and elderly (Hilgenkamp et al., 2012; Phillips & Holland, 2011). In the current study, adaptive behaviour, as an indicator for level of intellectual disability, explained a part of the cpm‐variance in the second model of the regression analysis: higher adaptive behaviour was associated with lower intensity. This is in contradiction to previous research, which showed that that physical activity levels seem to decrease with increasing severity of intellectual disability in adults and elderly (Hilgenkamp et al., 2012; Phillips & Holland, 2011). The difference could be explained by the difference between youth and adults. Children and adolescents with intellectual disability are dependent on their parents or caregivers, while in the adult population, individuals with less severe levels of intellectual disability are more independent and thereby more physically active to perform the activities of daily living. Another potential explanation of the unexpected finding is that our sample consisted of children and adolescents with moderate‐to‐severe intellectual disability attending specialized day program centres. The day activity programmes are likely different from those for children and adolescents attending a regular of special education school. Further research focusing on the relation between physical activity and level of intellectual disability in children and adolescents with intellectual disability is clearly needed, to effectively target interventions.

The fact that adaptive behaviour became a significant predictor in the second model could be caused by the overlap in explained variance between adaptive behaviour and motor development. Both motor development and adaptive behaviour are complicated constructs that are linked to specific brain structures and are interrelated (Diamond, 2000). Therefore, more research is required to study the relationship between adaptive behaviour, motor development and physical activity in children and adolescents with intellectual disability.

### 4.1 Strengths and limitations

The present authors studied physical activity levels by use of accelerometry in a specific group of children and adolescents that has hardly been studied. As far as the present authors know, this is the first study that used intellectual disability‐specific cut‐points to classify intensity of physical activity, and the first that assessed the relation between motor development and physical activity in children and adolescents with intellectual disability.

Some limitations do apply to this study. First, limitations are inherently associated with using accelerometers to study physical activity. Accelerometers are the preferred option to objectively study physical activity in children and adolescents with and without intellectual disability (Hinckson & Curtis, 2013; Reilly et al., 2008; Warren et al., 2010). However, activities like swimming are not registered by accelerometers. Parents could note these events on standardized sheets, but these were rarely completed. Therefore, an underestimation of the volume of physical activity could have occurred.

Furthermore, the intellectual disability‐specific cut‐points used in the current study (McGarty et al., 2016) were developed in children aged 8 to 11 years. These cut‐points were not studied in children with other ages; therefore, the present authors cannot be sure under‐ or overestimation has occurred in our participants with other ages. However, based on the study of Trost, Loprinzi, Moore, and Pfeiffer (2011) which indicated that Evensons’ cut‐points (developed for TD children age 5–8 years) were suitable for older TD children and adolescents (5–15 years) as well, the present authors think this effect is likely to be small.

In the current study, only 59% (68/115) participants receiving an accelerometer had enough data to be included in the analyses. Eleven participants did not wear the accelerometer at all. In previous research in children and adolescents with intellectual disability, compliance rates of 50%–100% were seen (Leung et al., 2017). The present authors asked the parents or caregivers to complete a sheet in case of special events, and more effort was potentially needed to increase compliance. The dropout, however, did not seem to be selective, so it did not influence the generalizability of our results to children and adolescents with moderate‐to‐severe intellectual disability, attending specialized day programme centres. However, generalizability to other groups of children and adolescents is not appropriate.

With regard to the statistics, the present authors used relatively many predictors in the regression models. The risk of putting too many predictors in the model is that small effects are difficult to detect. Furthermore, the present authors found small explained variance in the models. Therefore, a large portion of the variance in physical activity outcomes is caused by other variables. Further research is necessary to study the nature of these variables.

Lastly, the current study had a cross‐sectional design. It gives insight into possible associations between physical activity behaviour and child characteristics, but not into causality of motor development for physical activity. Longitudinal studies are required to study this relationship over time.

### 4.2 Implications for research and practice

Future research should focus on the effectiveness of interventions to promote physical activity and motor development in children and adolescents with moderate‐to‐severe intellectual disability, and the transfer of physical activity and its benefits over time in this population. Furthermore, raw data of accelerometers should be included in the manuscripts.

Policy makers, therapists, parents and caregivers should pay more attention to improve the physical activity behaviour in children and adolescents with moderate‐to‐severe intellectual disability attending specialized day programme centres. Attention is needed for the volume of habitual physical activity, but also on the quality of physical activity. It seems important to stimulate the development of motor skills, in order to increase the volume of physical activity now and in the future. In this field, professionals in the field of adapted physical activity and education can play exquisite pivotal role.

## 5 CONCLUSION

More than half of the participants were not meeting the physical activity recommendation of minimal 60‐min MVPA per day. Family and caregivers of these children should focus on supporting and motivating them to explore and expand their physical activities.

## APPENDIX 1

### **Table A1** Descriptive statistics of physical activity parameters sorted by boys vs. girls, and diagnosed with Down syndrome or not (*M* ± *SD*, [95% CI])

**Table:**

	Boys		Girls		No DS		DS	
	*M* ± *SD*	95% CI	*M* ± *SD*	95% CI	*M* ± *SD*	95% CI	*M* ± *SD*	95% CI
*N*	43		25		52		16	
Valid days (*n*)	6.6 ± 1.3	6.2–7.0	6.2 ± 1.3	5.7–6.7	6.3 ± 1.3	6.0–6.7	6.9 ± 1.4	6.2–7.6
Wear time (min)	683 ± 80	658–707	661 ± 67	633–689	671 ± 64	653–689	687 ± 109	629–745
Steps per day	7,257 ± 2,554	6,471–8,043	5,680 ± 2,412	4,685–6,676	6,865 ± 2,758	6,097–7,633	6,067 ± 1,951	5,028–7,107
Counts per minute	1,070 ± 364	958–1,182	988 ± 531	769–1,207	1,030 ± 464	900–1,159	1,074 ± 310	908–1,239
Minutes MVPA^a^	101 ± 44	87–114	77 ± 46	58–97	88 ± 47	75–102	104 ± 39	83–124
Daily ≥60 min MVPA^a^	26 (60%)	45–76	6 (24%)	6–42	24 (46%)	32–60	8 (50%)	22–78
Distribution throughout the day(%)^a^								
Sedentary	77 ± 9	74–80	81 ± 9	77–84	79 ± 9	76–81	77 ± 7	73–81
Light PA	8 ± 3	7–9	7 ± 2	7–8	8 ± 3	7–9	8 ± 2	7–9
Moderate PA	10 ± 4	8–11	7 ± 3	6–9	9 ± 4	7–10	10 ± 4	8–11
Vigorous PA	5 ± 3	4–6	4 ± 5	2–6	5 ± 4	4–6	6 ± 3	4–7

Notes

DS, down syndrome; PA, physical activity; MVPA, moderate‐to‐vigorous physical activity. *Group difference (*χ* ^2^(1)* = *8,438; *p* = 0.004).^a^Intensity based on McGarty's VM‐cut‐points.
